# FeCl_3_ as a low-cost and efficient p-type dopant of Spiro-OMeTAD for high performance perovskite solar cells†

**DOI:** 10.1039/c8ra00243f

**Published:** 2018-03-05

**Authors:** Xiaoyu Gu, Yang Li, Yanfei Mu, Min Zhang, Tongbu Lu, Peng Wang

**Affiliations:** Institute for New Energy Materials & Low-Carbon Technologies, Tianjin University of Technology Tianjin 300384 China zm2016@email.tjut.edu.cn; Department of Chemistry, Zhejiang University Hangzhou 310028 China; Changchun Institute of Applied Chemistry, Chinese Academy of Sciences Changchun 130022 China

## Abstract

Chemical doping is a viable tactic to improve the charge transporting properties of organic semiconductors in efficient perovskite solar cells. In this paper, we first employ the low-cost inorganic salt FeCl_3_ as a chemical dopant to replace the traditional expensive cobalt complex for the oxidization of 2,2′,7,7′-tetrakis(*N*,*N-p*-dimethoxyphenylamino)-9,9′-spirobifluorene (Spiro-OMeTAD), a typical organic hole-transporter. Based on the joint measurements of electron absorption spectra, cyclic voltammetry, and the hole-only device, we reveal that FeCl_3_ can effectively oxidize Spiro-OMeTAD and improve the hole transporting properties of Spiro-OMeTAD. Through carefully optimizing the dopant concentration, solar cells with 80% FeCl_3_ doped Spiro-OMeTAD achieve over 17% power conversion efficiency based on a triple cation perovskite photoactive layer, which is comparable to that of devices with a classical cobalt complex dopant. Our work demonstrates the potential of using FeCl_3_ as a low-cost chemical dopant for hole-transporting materials in perovskite solar cells.

## Abstract 2

We have employed low-cost FeCl_3_ as efficient dopant of Spiro-OMeTAD for high performance perovskite solar cells.

## Graphical abstract

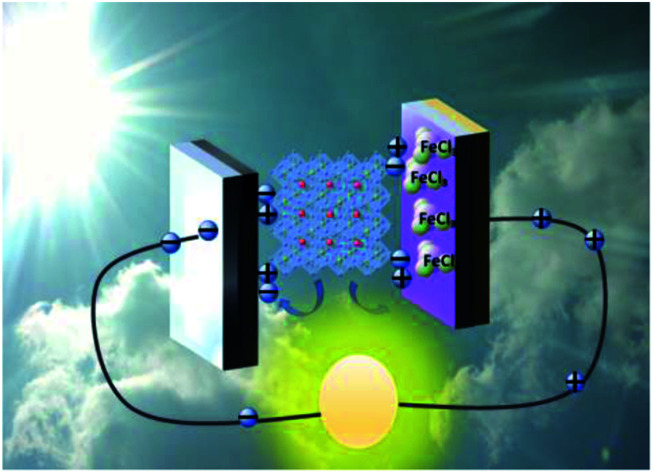

## Introduction

Organic–inorganic hybrid perovskite solar cells (PSCs) have gone through an unexpected rapid development in the past six years since the introduction of solid organic semiconductors as a hole-transporting material (HTM).^1–11^ In a typical perovskite solar cell, a perovskite photoactive layer (ABX_3_, A = CH_3_NH_3_, CH_3_(NH_2_)_2_, or Cs; B = Pb or Sn; X = Cl, Br, or I) is sandwiched between an electron-transporting material (ETM) and a HTM. Carriers are generated in the perovskite layer upon the absorption of photons. Subsequently, photogenerated electrons and holes traverse through several hundred nanometers-thick charge transporting materials ETM and HTM, respectively, and come to the current collectors. Charge transporting materials featuring good carrier transporting properties are critically important to achieve high performance in PSCs.^12^

Spiro-OMeTAD is the mostly widely used HTM among all the top performing PSCs.^10,11^ Owing to the relatively low hole mobility of Spiro-OMeTAD (∼10^−5^ cm^2^ V^−1^ s^−1^),^13,14^ many additives, *i.e.* lithium bis(trifluoromethanesulfonyl)imide (LiTFSI), 4-*tert*-butylpyridine (TBP), are always added together with Spiro-OMeTAD during film processing to achieve a better device performance. LiTFSI was reported to dramatically improve the electrical conductivity and hole mobility of Spiro-OMeTAD^14^ while TBP can effectively restrain the charge recombination and function as a morphological controller for the hole-transporting layer.^15–17^ Snaith *et al.* has demonstrated that LiTFSI assists the oxidization reaction between Spiro-OMeTAD and O_2_ in the surrounding atmosphere,^18^ which shows atmospheric dependencies leading to the variable results and poor reproducibility. To move towards more stable and reproducible devices, N(PhBr)_3_SbCl_6_,^19^ F4-TCNQ,^20^ SnCl_4_,^21^ AgTFSI,^22^ CuI^23^ and cobalt-complexes (such as FK102 (ref. 24) and FK209 (ref. 10 and 25)) have been reported as the chemical dopants for the oxidization of Spiro-OMeTAD. FK209 shows the best device performance among all the oxidizing agents. However, FK209 is quite expensive due to the complexity of synthesis and the requirements of high purity. Hence, it is really pertinent to identify a low-cost and efficient chemical dopant for the oxidation of Spiro-OMeTAD.

In this work, we first report FeCl_3_ as a low-cost and efficient chemical dopant for Spiro-OMeTAD to obtain a high performance perovskite solar cell. Our original motivation for choosing FeCl_3_ as the oxidizing agent is the more positive redox potential of Fe^3+^/Fe^2+^ (NHE, 0.771 V) relative to that of Spiro-OMeTAD^+^/Spiro-OMeTAD (NHE, 0.63 V (ref. 26)). The 0.14 V potential gap shows the feasibility in thermodynamics of the oxidation reaction Fe^3+^ + Spiro-OMeTAD → Fe^2+^ + Spiro-OMeTAD^+^. In the preliminary experiment, it was found that the Spiro-OMeTAD solution shows light yellow, and after adding a small amount of FeCl_3_ dopant, the colour turns to dark purple immediately (Fig. S1 in the ESI†). The colour change indicates a chemical reaction may happen after introducing FeCl_3_, instead of a physical mixture. In this contribution, through joint photophysical and electrical studies, we will take a close look at the origins of FeCl_3_ on the transporting property of Spiro-OMeTAD.

## Results and discussion

**Fig. 1:**
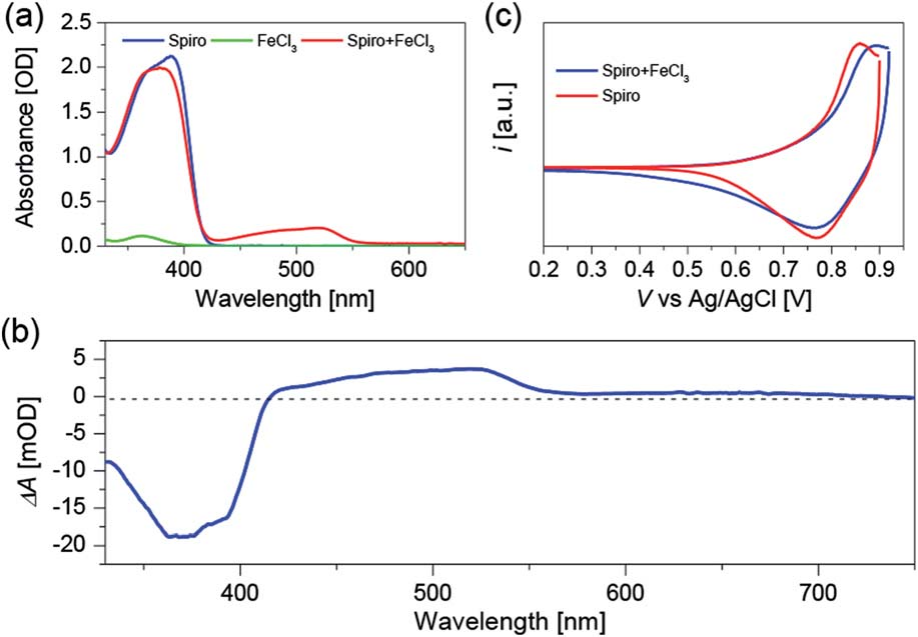
(a) Absorption spectra of Spiro-OMeTAD (abbreviated as Spiro) in chlorobenzene (30 mM), FeCl_3_ in chlorobenzene/acetonitrile (v/v, 1/1) mixture (24 mM), Spiro-OMeTAD (30 mM) and FeCl_3_ (24 mM) (abbreviated as Spiro + FeCl_3_) dissolved in chlorobenzene/acetonitrile (v/v, 1/1) mixture. (b) Wavelength-dependent absorption change upon applying a positive potential bias to a Spiro-OMeTAD deposited FTO film. (c) CV of Spiro-OMeTAD (abbreviated as Spiro) and FeCl_3_ doped Spiro-OMeTAD (abbreviated as Spiro + FeCl_3_) deposited on FTO film immersed in EMITFSI as the supporting electrolyte. CV was measured using a three-electrode system, Pt as the counter electrode, and Ag/AgCl as the reference electrode. Scan rate: 10 mV s^−1^. The onset potential is 0.75 V for Spiro-OMeTAD, and 0.77 V for FeCl_3_ doped Spiro-OMeTAD. The oxidation potential is 0.86 V for Spiro-OMeTAD, and 0.89 V for FeCl_3_ doped Spiro-OMeTAD.

To clarify the validity of the oxidation capability of FeCl_3_, we firstly measured the electronic absorption spectra of Spiro-OMeTAD dissolved in chlorobenzene with or without FeCl_3_ doping as shown in Fig. 1a. The peak at around 390 nm is ascribed to the absorption of Spiro-OMeTAD, and a new absorption band with the peak maximum located at 520 nm appears after introducing FeCl_3_ into Spiro-OMeTAD. FeCl_3_ itself does not feature absorption around 520 nm, hence, the new absorption band of the Spiro-OMeTAD/FeCl_3_ mixture can be ascribed to the formation of oxidized Spiro-OMeTAD radical, which has been proved when using O_2_ (ref. 27) or cobalt complex^25^ as chemical dopants for Spiro-OMeTAD. All the measurements were carried out under N_2_ atmosphere to avert the oxidation of Spiro-OMeTAD by O_2_. The additional peak at 520 nm uncovers the occurrence of charge transfer reaction between Spiro-OMeTAD and FeCl_3_. With respect to the intensity at 390 nm of pristine Spiro-OMeTAD, the decreased intensity at this wavelength for Spiro-OMeTAD/FeCl_3_ mixture can be attributed to the decreased amount of Spiro-OMeTAD due to the oxidation reaction between Spiro-OMeTAD and FeCl_3_.

To further confirm the origin of the absorption peak at 520 nm, we conducted the spectroelectrochemical measurement (Fig. 1b) by using a Spiro-OMeTAD film without any dopants. Spiro-OMeTAD deposited FTO film and Pt wire are connected to the CHI660D electrochemical workstation to supply the potential bias, and 1-ethyl-3-methylimidazolium bis(trifluoromethanesulfonyl)imide (EMITFSI) ionic electrolyte is used as the supporting electrolyte. After applying a positive potential bias to the Spiro-OMeTAD film, we can see a positive absorption change at 520 nm, which is probably originated from the absorption of Spiro-OMeTAD^+^ produced by hole injection from external circuit. Moreover, the negative signal at around 390 nm can be attributed to the photo-bleaching of Spiro-OMeTAD. Furthermore, we performed the cyclic voltammetry (CV) measurements as shown in Fig. 1c to explore the influence of FeCl_3_ dopant on the highest occupied molecular orbital (HOMO) energy level of Spiro-OMeTAD. The CV measurements show that Spiro-OMeTAD has a more positive oxidation onset and peak potential after introducing FeCl_3_ as a chemical dopant, resulting in a stabilized HOMO energy level of FeCl_3_ doped Spiro-OMeTAD. The CV results evidently demonstrate chemical reaction between Spiro-OMeTAD and the FeCl_3_ dopant.

**Fig. 2:**
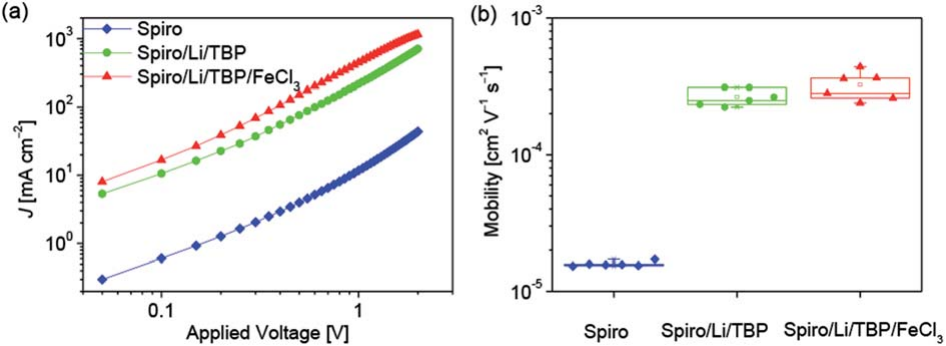
(a) Double logarithmic *J*–*V* curves measured under dark of hole only device based on pristine Spiro-OMeTAD (coded as Spiro), LiTFSI and TBP doped Spiro-OMeTAD (coded as Spiro/Li/TBP) as well as LiTFSI, TBP and FeCl_3_ doped Spiro-OMeTAD (code as Spiro/Li/TBP/FeCl_3_). (b) Hole mobility of Spiro-OMeTAD with or without dopants. The thickness of the HTM films is kept the same (80 nm).

According to the equation *σ* = *nqμ*, where *σ*, *n*, *q* and *μ* are the electrical conductivity, concentration, charge number and mobility, respectively. The increase in the concentration of oxidized Spiro-OMeTAD probably leads to improvement of electrical conductivity due to the increased hole concentration. In order to investigate the effect of FeCl_3_ dopant on the electrical conductivity of Spiro-OMeTAD, hole only devices with the structure of ITO/PEDOT:PSS/Spiro-OMeTAD (with or without dopants)/Au were fabricated. Fig. 2a shows the current density–voltage (*J*–*V*) double logarithmic characteristic curves of three hole only devices using pure Spiro-OMeTAD, Spiro-OMeTAD doped with LiTFSI and TBP, Spiro-OMeTAD doped with LiTFSI, TBP and FeCl_3_ as HTMs. During the device fabrication, the thickness of Spiro-OMeTAD film and the cell area have been kept the same, hence the increase current density at a certain voltage is related to the improved electrical conductivity according to the equation *σ* = *L*/*RA* = *JL*/*VA*, where *R*, *L*, and *A* are the resistance, length and cross-sectional area, respectively. As depicted in Fig. 2a, an over one order of magnitude larger current density for the LiTFSI and TBP doped Spiro-OMeTAD film is measured compared to the pure Spiro-OMeTAD at a given applied voltage, indicating a significant enhancement of electrical conductivity. Moreover, the electrical conductivity of HTM continuously increases when the Spiro-OMeTAD is co-doped with LiTFSI, TBP and FeCl_3_. The variation of electrical conductivity induced by the FeCl_3_ shows a similar trend with FK209 doped Spiro-OMeTAD.^25^

The mobility was further studied using space charge limited current (SCLC) model.^28,29^ The value was extracted from the fitting line of the *J*–*V* data (Fig. S2 in the ESI†) using the Mott–Gurney equation^30^ as shown in Fig. 2b. The averaged hole mobility of pristine Spiro-OMeTAD is 1.6 × 10^−5^ cm^2^ V^−1^ s^−1^. And over one order larger averaged hole mobility is obtained for LiTFSI and TBP doped Spiro-OMeTAD (2.6 × 10^−4^ cm^2^ V^−1^ s^−1^), which agrees well with the previous reports.^14^ On the basis of LiTFSI and TBP dopants, the hole mobility of Spiro-OMeTAD is further improved to 3.2 × 10^−4^ cm^2^ V^−1^ s^−1^ along with the introduction of FeCl_3_ dopant. In addition, increasing FeCl_3_ doping concentration will result in an enhancement of hole mobility of Spiro-OMeTAD as observed in the measurement of doping concentration related mobility (Fig. S3†). We propose that the FeCl_3_ may induce a more compact packing of Spiro-OMeTAD or change the Spiro-OMeTAD's packing mode during the film processing, which probably account for the improved hole mobility. Hence, the improved electrical conductivity for FeCl_3_ doped Spiro-OMeTAD (Fig. 2a) is related to both the increased concentration of oxidized Spiro-OMeTAD and the improved hole mobility.

**Fig. 3:**
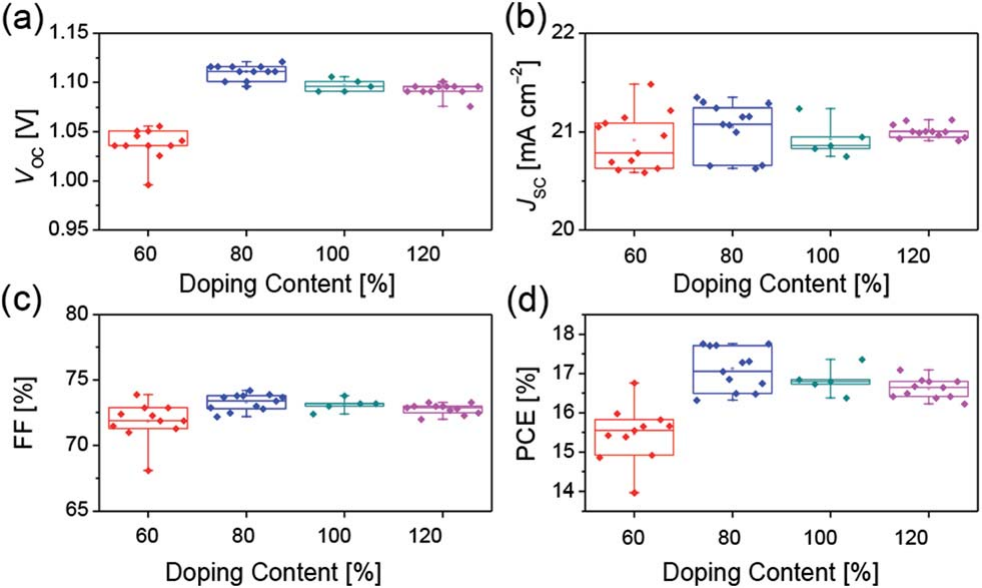
Device parameters (a) *V*_OC_, (b) *J*_SC_, (c) FF, (d) PCE of perovskite solar cells with different concentration of FeCl_3_ dopants.

The increased electrical conductivity of Spiro-OMeTAD using FeCl_3_ as the chemical dopant can probably lead to an improved device performance, which has been demonstrated in the following device studies. We investigated the device parameters (Fig. 3) as a function of the FeCl_3_ doping concentration based on the device structure FTO/compact-TiO_2_/mesoporous-TiO_2_/perovskite/Spiro-OMeTAD/Au. All the measurements were recorded in N_2_ atmosphere to avoid the oxidization of Spiro-OMeTAD by O_2_. As shown in Fig. 3a, the average open-circuit voltage (*V*_OC_) increases with doping concentration of FeCl_3_, which reaches to the maximum value of 1.09 V at 80% doping concentration, which benefits the improvement of the charge transport properties. And, further increase of FeCl_3_ doping concentration results in the decrease in *V*_OC_. No obvious variation tendency was found in short-circuit current density (*J*_SC_) and fill factor (FF) (Fig. 3b and c). We believe that the decreased *V*_OC_ could be ascribed to the increased charge recombination at the TiO_2_/perovskite/HTM interfaces. Although the hole mobility of Spiro-OMeTAD increases with increasing doping concentration of FeCl_3_ (Fig. S3 and Table S1†), which is favorable for the charge transporting of HTM, FeCl_3_ can effectively oxidize Spiro-OMeTAD and lower the HOMO energy level of HTM (Fig. 1c), leading to increasing hole concentration in HTM and decreasing driving force for hole extraction. Therefore, about 80% doping may be the equilibrium point for hole extraction and hole transporting as well as the interfacial recombination. Hence the best PCE (Fig. 3d) was obtained at 80% doping concentration of FeCl_3_.

**Fig. 4:**
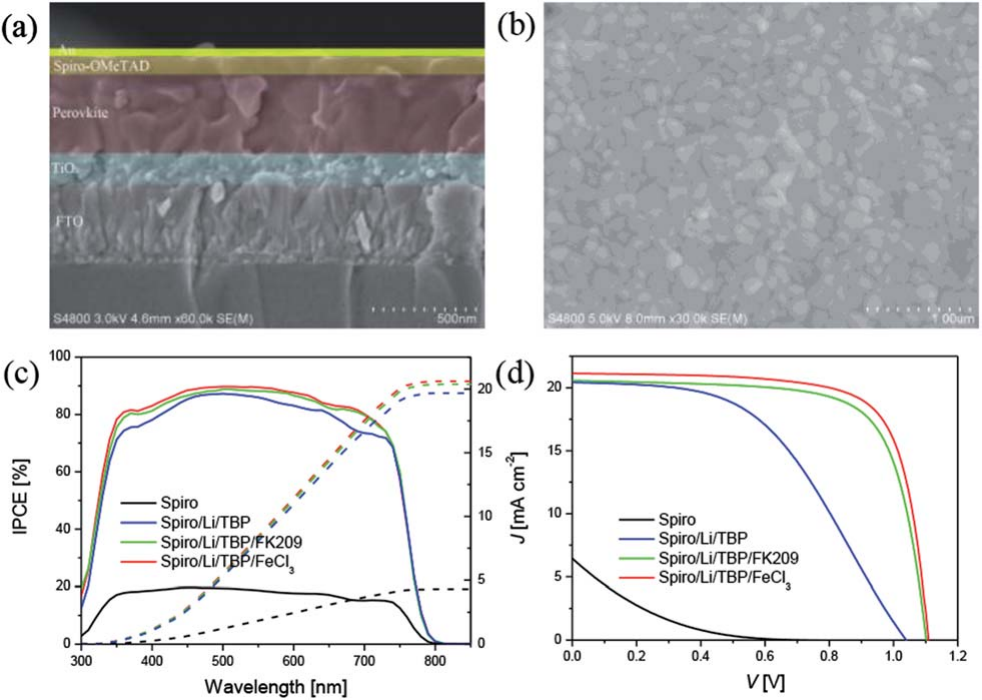
(a) Cross-section of perovskite solar cells with the structure FTO/TiO_2_/perovskite/Spiro-OMeTAD/Au. (b) SEM of the perovskite films. (c) IPCE and (d) *J*–*V* curves of devices based on different HTMs. The *J*–*V* curves were scanned at speed of 10 mV s^−1^.

Fig. 4a shows the cross-section of perovskite device, the thickness of all functional layers can be clearly recognized, ∼160 nm for oxide substrate, ∼380 nm for perovskite layer and ∼90 nm for HTM. The perovskite film shows a good film quality with a compact and uniform morphology (Fig. 4b), and it also features a granular structure with the grain size about several hundred nanometers. Fig. 4c and d summarized the wavelength dependent incident photo-to-collected electron efficiencies (IPCEs) and the *J*–*V* curves of perovskite solar cells with different HTMs. Devices with no HTM dopants are made as control, and the photovoltaic parameters of these devices are listed in Table 1. The control device behaves very poor performance due to the low hole mobility of pure Spiro-OMeTAD. After adding LiTFSI and TBP to Spiro-OMeTAD, the *V*_OC_, *J*_SC_ and FF are significantly improved, generating a power conversion efficiency (PCE) of 10.3%. Moreover, when oxidizing agent FK209 or FeCl_3_ was employed as chemical dopants, FF and *V*_OC_ were further improved due to the improved electrical conductivity and the decreased HOMO energy level of HTM. It is noted that the *J*_SC_, *V*_OC_ and FF of low-cost FeCl_3_ based device are 21.14 mA cm^−2^, 1.11 V and 0.734, affording a comparable PCE of 17.2% with respect to that of device with traditional FK209 dopant (16.2%).

**Table 1:** Photovoltaic parameters under 100 mW cm^−2^ stimulated AM1.5G sunlight^a^

HTM	*J* ^IPCE^_SC_ [mA cm^−2^]	*J*_SC_ [mA cm^−2^]	*V*_OC_ [V]	FF	PCE [%]
Spiro	4.31	6.44 ± 0.72	0.70 ± 0.06	0.124 ± 0.064	0.6 ± 0.3
Spiro/Li/TBP	19.67	20.42 ± 0.65	1.04 ± 0.04	0.484 ± 0.046	10.3 ± 1.5
Spiro/Li/TBP/FK209	20.36	20.57 ± 0.48	1.10 ± 0.01	0.718 ± 0.010	16.2 ± 0.6
Spiro/Li/TBP/FeCl_3_	20.61	21.14 ± 0.42	1.11 ± 0.01	0.734 ± 0.011	17.2 ± 0.7

a Each value is the average of the data taken from 12 devices. *J*^IPCE^_SC_ was derived *via* wavelength integration of the product of the standard AM1.5G emission spectrum (ASTM G173-03) and the IPCEs were measured at the short-circuit.

## Conclusions

To summarize, we have reported a high efficiency perovskite solar cells using FeCl_3_ as a cost-effective p-type dopant for Spiro-OMeTAD. The electronic absorption spectra and spectroelectrochemical results revealed the presence of oxidization reaction between FeCl_3_ dopant and Spiro-OMeTAD. The CV results showed that the HOMO energy level of Spiro-OMeTAD was stabilized after adding FeCl_3_ dopants. The *J*–*V* measurements of hole-only device have told us that the increased hole concentration and mobility jointly results in the improved electrical conductivity of FeCl_3_ doped Spiro-OMeTAD. The high efficiency perovskite solar cells exhibiting over 17% PCE was obtained when employing FeCl_3_ as dopants due to the improved hole transporting ability. Our work has demonstrated the commercial potential for the application of FeCl_3_ as the p-type dopants.

## Experimental section

### Deposition of compact layer and mesoporous layer

Pre-etched fluorine-doped tin oxide (FTO) coated glass substrates were cleaned using detergent and sequentially ultrasonicated in deionized water, acetone and ethanol (10 min each). After drying, the substrates were further cleaned in UV–ozone for 15 min. To form a compact TiO_2_ electron-transporting layer, FTO substrates were immersed into the 40 mM TiCl_4_ aqueous solution at 70 °C for 90 min, then rinsed with deionized water and annealed at 450 °C for 30 min. After cooling down to room temperature, a mesoporous TiO_2_ layer was deposited on the compact TiO_2_ layer through spin coating for 20 s at 4000 rpm using TiO_2_ paste (30NR-D, Dyesol) diluted with anhydrous ethanol at a weight ratio of 1 : 6. The substrates were then sequentially annealed at 100 °C for 10 min and sintered at 450 °C for 30 min.

### Preparation of the perovskite precursor solution and deposition progress

Perovskite precursor was prepared as previously reported:^31^ FAI (Xi'an Polymer Light Technology Corp., ≥99.5%), PbI_2_ (TCI, 99.99%), MABr (Xi'an Polymer Light Technology Corp., ≥99.5%) and PbBr_2_ (aladdin, 99.999%) were mixed with the molar concentration of 1 M, 1.1 M, 0.2 M, and 0.2 M in anhydrous DMF (Sigma Aldrich, 99.8%) : DMSO (Sigma Aldrich, 99.9%) 4 : 1 (v/v) solution. 1.5 M CsI (aladdin, 99.999%)/DMSO stock solution added into the perovskite precursor to obtain the desired triple cation composition. The perovskite precursor was stirred in a nitrogen-filled glove box to fully dissolve the chemical components and then filtered with a PTFE filter (pore diameter: 0.22 μm) right before using. The deposition process contains a two-step spin coating program with a speed of 1000 rpm for 10 s and 6000 rpm for 20 s respectively. 150 μL chlorobenzene was dripped onto the substrate 5 s prior to the termination of the program. The substrates were then annealed at 100 °C for 1 h under N_2_ atmosphere.

### Deposition of the FeCl_3_ doped Spiro-OMeTAD and the top electrode

60 mM Spiro-OMeTAD (Xi'an Polymer Light Technology Corp., ≥99.8%) solution was obtained by dissolving 72.3 mg Spiro-OMeTAD, 28.8 μL TBP and 17.5 μL LiTFSI stock solution (520 mg mL^−1^ in acetonitrile) into 1 mL of chlorobenzene. FeCl_3_ (Alfa Aesar, 98%) solution (100 mg mL^−1^ in acetonitrile) was added into Spiro-OMeTAD solution to achieve the desired doping concentration. The hole transporting layer was deposited atop perovskite substrate through spin coating above mentioned solutions at 6000 rpm for 30 s. Finally, a 80 nm-thick Au top electrode was thermally evaporated onto substrates. For FK209 doping device, the film was deposited by spin coating the mixed solution of 68.6 μL FK209 solution (300 mg mL^−1^ in acetonitrile) and 1 mL Spiro-OMeTAD solution.

### SEM, UV-vis, and spectroelectrochemical measurements

Scanning electron microscopic images were recorded with a Hitachi S-4800 Instrument (Japan). UV-vis absorption spectra were recorded on a Perkin Elmer Lambda 900 spectrophotometer. For the spectroelectrochemical measurements, CHI660C electrochemical workstation was used to supply the potential bias, and the spectra were recorded on a Perkin Elmer Lambda 900 spectrophotometer. The measurements were conducted as follows: First, the ground state Spiro-OMeTAD film was taken as the background. Then, a small positive potential bias was applied to the film using chronoamperometry method to produce the oxidation state of Spiro-OMeTAD, and a spectrum was obtained after 1 min. The absorption change reflects the species change on the film after applying the potential bias.

### IPCE and *J*–*V*

The IPCE measurements were performed with a system combining a 150 W xenon lamp, a Zolix Omni-*λ*200i monochromator, a Keithley 2400 source meter and a Hamamatsu S1337-1010BQ silicon diode calibrated in National Institute of Metrology, China. The data were collected with a wavelength sampling interval of 10 nm and a current sampling time of 1 s with full computer control. The cell performance evaluated by the *J*–*V* measurements under simulated solar light (a model LS1000-4S-AM1.5G-1000 W solar simulator, Solar Light Company, USA) with a light intensity of 100 mW cm^−2^, tested with a PMA2144 pyranometer and a calibrated PMA 2100 dose control system. The effective area was set to 0.04 cm^2^ by using a metal mask.

## Conflicts of interest

There are no conflicts to declare.

## Supplementary Material

RA-008-C8RA00243F-s001
