# Supplementary material for: FeCl3 as a low-cost and efficient p-type dopant of Spiro-OMeTAD for high performance perovskite solar cells
Source: RSC Adv. 2018 Mar 5;8(17):9409–13. doi: 10.1039/c8ra00243f (PMC9078656; doi:10.1039/c8ra00243f)
Supplement: RA-008-C8RA00243F-s001 [file RA-008-C8RA00243F-s001.pdf]

Electronic Supplementary Material (ESI) for RSC Advances.
This journal is © The Royal Society of Chemistry 2018

Electronic supplementary information

FeCl$_3$ as a low-cost and efficient p-type dopant of Spiro-OMeTAD for high performance perovskite solar cells†

Xiaoyu Gu,[a,b] Yang Li,[b,c] Yanfei Mu,[a,b] Min Zhang,*[a] Tongbu Lu[a] and Peng Wang[b]

[a] *Institute for New Energy Materials & Low-Carbon Technologies, Tianjin University of Technology, Tianjin, 300384, China. Email: zm2016@email.tjut.edu.cn.*

[b] *Department of Chemistry, Zhejiang University, Hangzhou, 310028, China.*

[c] *Changchun Institute of Applied Chemistry, Chinese Academy of Sciences, Changchun, 130022, China.*

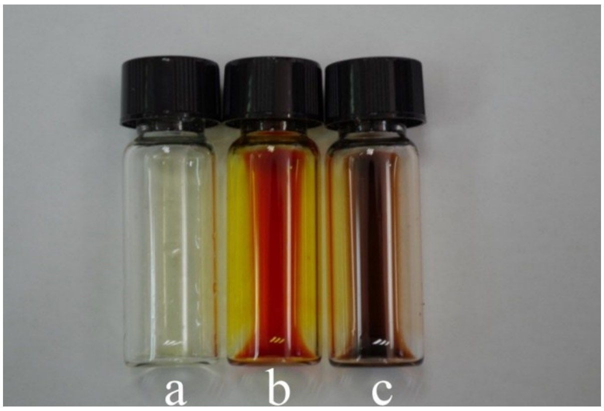

Fig. S1 Images of solutions: (a) Spiro-OMeTAD in chlorobenzene (60 mM), (b) $FeCl_3$ in acetonitrile (100 mg/ml), and (c) adding solution b (8 μL) into solution a (100 μL).

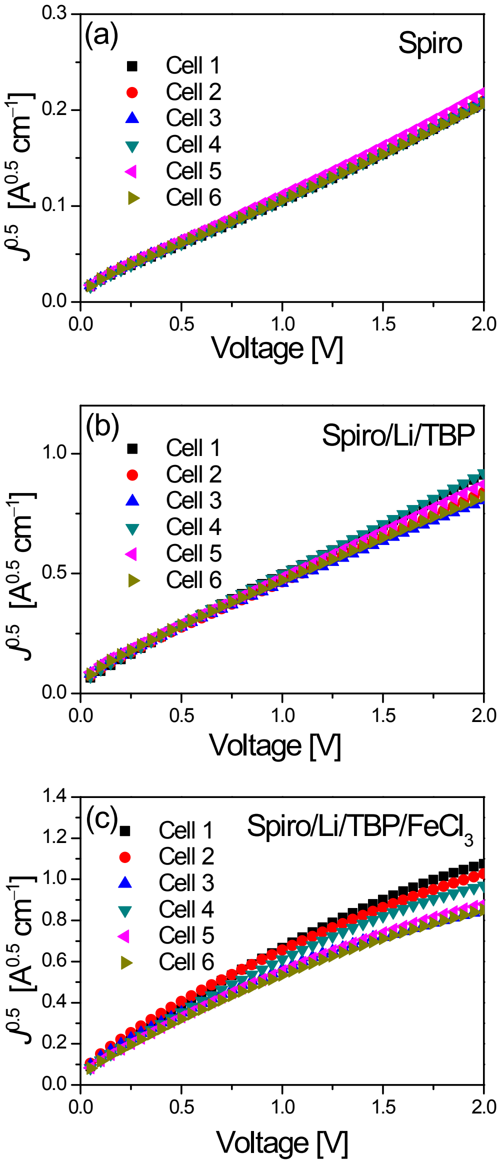

Fig. S2 Root of current–voltage ($J^{0.5}$–V) curves with the hole-only device using (a) pure Spiro-OMeTAD (coded as Spiro), (b) Spiro-OMeTAD doped with LiTFSI and TBP (coded as Spiro/Li/TBP), (c) Spiro-OMeTAD doped with LiTFSI, TBP and $FeCl_3$ (coded as Spiro/Li/TBP/$FeCl_3$).

The mobility (μ) was obtained through the fitting line of the J-V curves using Mott-Gurney equation,

$$J = \frac{9}{8}\varepsilon_0\varepsilon_r\mu\frac{V^2}{d^3}.$$

Here, ε_0 is the vacuum permittivity with the value of 8.85×10^{-12} F/m. ε_r is the dielectric constant of the materials, which has a value of 3 for most organic semiconductors. d is the film thickness (80 nm).

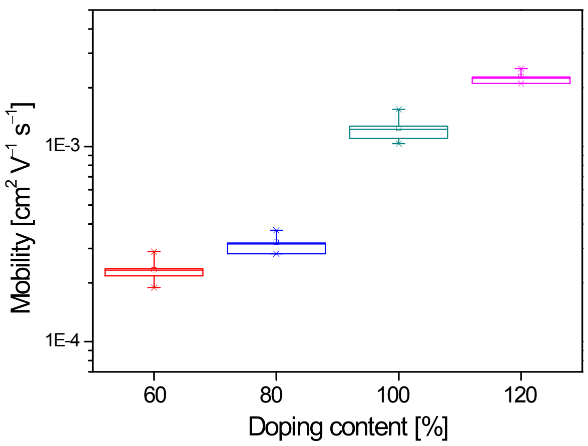

Fig. S3 Scatter diagram of mobility for Spiro-OMeTAD at doping concentration of 60%, 80%, 100%, 120%.

Table S1 Summarized data for hole mobility of Spiro-OMeTAD as a function of doping concentration

Doping concentration (%)	Hole mobility μ ($cm^2\ V^{-1}\ S^{-1}$)
60	2.6×10^{-4}
80	3.2×10^{-4}
100	1.2×10^{-3}
120	2.3×10^{-3}
